# The Effect of Cationic Polyamidoamine Dendrimers on Physicochemical Characteristics of Hydrogels with Erythromycin

**DOI:** 10.3390/ijms160920277

**Published:** 2015-08-27

**Authors:** Magdalena Wróblewska, Katarzyna Winnicka

**Affiliations:** Department of Pharmaceutical Technology, Faculty of Pharmacy, Medical University of Białystok, Mickiewicza 2c, 15-222 Białystok, Poland; E-Mail: magdalena.wroblewska@umb.edu.pl

**Keywords:** PAMAM dendrimer, erythromycin, hydrogel, mechanical properties, rheological properties

## Abstract

Polyamidoamine dendrimers (PAMAM) represent a new class of hyperbranched, monodisperse, three-dimensional polymers with unique properties, which make them very promising carriers of antimicrobial agents. The present study aimed to evaluate the influence of PAMAM-NH_2_ dendrimers generation two (G2) or three (G3) on physicochemical characteristics and structure of hydrogels with a model antibacterial lipophilic drug—erythromycin—commonly used in topical applications. From the obtained rheograms, it can be concluded that tested hydrogels were non-Newtonian thixotropic systems with shear-thinning behaviour. The dissolution tests revealed that erythromycin was definitely faster released from formulations containing PAMAM-NH_2_ in concentration and generation dependent manner. However, the addition of PAMAM-NH_2_ to hydrogels evoked only slight improvement of their antibacterial activity. It was also shown that the structure of hydrogels changed in the presence of PAMAM-NH_2_ becoming less compact, diversified and more porous. Designed hydrogels with PAMAM-NH_2_ G2 or G3 were stable stored up to three months at 40 ± 2 °C and 75% ± 5% RH.

## 1. Introduction

Topical therapy is an important treatment option due to its non-invasiveness, ease of use, no risk of systematic adverse effects or drug interactions and better patient compliance. Topical formulations therapeutic efficacy depends on both the physicochemical properties of an active compound and the type of the vehicle. Currently, numerous studies have highlighted the pharmaceutical importance of hydrogels as vehicles for dermal delivery of a wide variety of drugs [1]. Hydrogels are three-dimensional and hydrophilic polymer networks capable of swelling in water or biological fluids and retaining a large amount of fluids in the swollen state [2]. Dermatological use of hydrogels have several favourable features *i.e.*, biocompatibility, adhesiveness, thixotropy, spreadability or simplicity of removal. Moreover, hydrogels are characterized by ease of application and better percutaneous absorption than other semisolid preparations. They provide faster and more complete release of the drug from the vehicle to the skin and as the consequence, higher efficacy than creams or ointments [1,2,3]. Dendrimers are hyperbranched, monodisperse polymers with precisely defined molecular weight and host-guest entrapment properties. Polyamidoamine dendrimers (PAMAM) are the most widely studied and characterized class of dendrimers. PAMAM are water-soluble, nonimmunogenic and biocompatible compounds, and their cytotoxicity is generation, surface charge and concentration dependent [4,5,6,7]. Due to their unique properties, PAMAM are considered as carriers for a variety of drugs including antibacterial or antifungal, with capacity to improve their solubility, therapeutic efficiency and drug permeation [8,9,10,11,12,13,14]. In addition, it was shown that amino-terminated PAMAM dendrimers possessed high antibacterial efficacy, which is attributed to the electrostatic interaction between the cationic dendrimer and the anionic bacterial cell surface with resultant disruption of the lipid bilayer and in the consequence cell lysis [14,15,16].

Erythromycin (EM) is a mixture of macrolide antibiotics produced by *Streptomyces erythreus*. EM is soluble in alcohol and slightly soluble in water [17] and is commonly used in topical applications for the treatment of acne vulgaris, acne rosacea, infections of skin and soft tissue, inflammation of the gums and eyelids. It acts primarily bacteriostatic against Gram(+) bacteria [18]. Commercial EM preparations for topical treatment are generally formulated as high alcohol content solutions (52%–92%) or petrolatum and mineral oil ointments. Ingredients present in these formulations have been reported as probable causes of adverse effects such as local dryness, oiliness, desquamation [19,20].

Research areas on the application of dendrimers in pharmaceutical technology are mostly limited to the simple physicochemical systems. Because, to our best knowledge, there are no studies describing how the addition of dendrimers affects rheological properties, texture and surface morphology of hydrogels, the aim of this study was to design hydrogels with EM and to evaluate the effect of PAMAM-NH_2_ generation 2 (G2) or 3 (G3) on their physicochemical characteristics. Moreover, the influence of PAMAM-NH_2_ on the *in vitro* EM release, antimicrobial activity and hydrogel stability was examined.

## 2. Results and Discussion

### 2.1. Characterization of Erythromycin Hydrogels with PAMAM-NH_2_ Generation 2 (G2) and Generation 3 (G3)

During designing dermal formulations, the choice of the appropriate vehicle as well as the addition of excipients plays an important role. It determines the quality of the dosage form, its stability and efficacy. In order to enhance drug transdermal absorption, chemical enhancers are used, which increased solubility of active substances in the vehicle or modify barrier properties of the stratum corneum [21]. One of the new advanced transdermal permeation-enhancement technologies is using nanocarriers such as dendrimers. Dendrimers interact with lipids presented at the cell membranes and facilitate diffusion of drugs into the skin. Moreover, dendrimers may act like solubility enhancers, increasing the permeation of lipophilic drugs [22,23]. In our previous study, it was demonstrated that PAMAM dendrimers significantly enhanced the solubility of EM in concentration dependent manner [24].

The present study focused on the influence of PAMAM-NH_2_ dendrimers G2 and G3 on physicochemical properties of hydrogels with EM (Table 1).

**Table 1:** Composition of prepared hydrogels.

Ingredient (g)	Formulation Code								
	E1	EP1	EP2	EP3	EP4	EP5	EP6	EP7	EP8
EM	2.0	2.0	2.0	2.0	2.0	2.0	2.0	2.0	2.0
HEC	2.5	2.5	2.5	2.5	2.5	2.5	2.5	2.5	2.5
Propylene glycol	10.0	10.0	10.0	10.0	10.0	10.0	10.0	10.0	10.0
Bronopol	0.02	0.02	0.02	0.02	0.02	0.02	0.02	0.02	0.02
PAMAM-NH_2_ G2	-	0.003	0.03	0.3	0.6	-	-	-	-
PAMAM-NH_2_ G3	-	-	-	-	-	0.003	0.03	0.3	0.6
Purified water (up to)	100	100	100	100	100	100	100	100	100

Prepared hydrogels were characterized by smooth, uniform consistency and they were easily spreadable. The presence of EM gave the preparations a white colour. The pH value of obtained hydrogels was in the range of 8.5–8.59 and was only a little higher compared with hydrogel without dendrimers. The stability of EM is pH dependent; at pH < 7.0, EM is unstable and the optimal pH for semisolid EM preparations is between 7–8.5 [25,26]. Therefore, obtained pH values should not affect its degradation. It was observed that the particle size of EM decreased with the increase of PAMAM-NH_2_ concentration, and it was probably caused by improved solubility of EM in the presence of dendrimers (Table 2).

**Figure 1:**
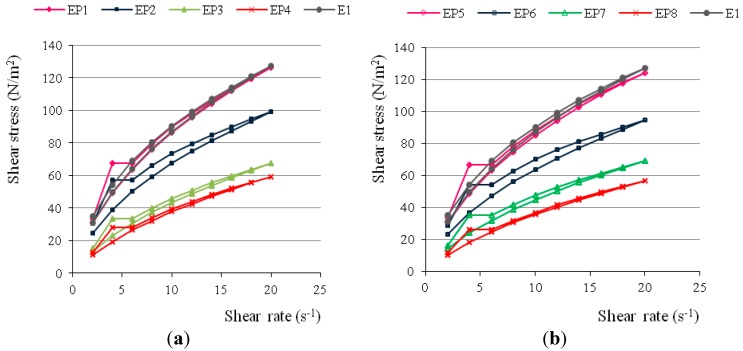
Rheograms of hydrogels with different concentrations of (**a**) PAMAM-NH_2_ G2 and (**b**) PAMAM-NH_2_ G3.

Knowledge of rheological behaviour of systems designed for topical application provides qualitative and quantitative information about the internal hydrogel structure. In addition, rheological determinations are important because of their contribution to the characterization of changes that may occur during storage. Rheological properties also relate to the spreading and application behaviour and patient acceptability. It was revealed that the viscosity of hydrogels declined with the growth in PAMAM-NH_2_ concentration. A marked reduction in viscosity (more than 2-fold) was noticed in the case of higher concentrations (3 and 6 mg/g) of PAMAM-NH_2_ G2 and G3 (*p* < 0.05). From the rheograms (Figure 1), it was deducted that tested hydrogels were non-Newtonian systems, they showed a shear-thinning behaviour and their viscosity decreased with growing shear rate. Examined hydrogels possessed thixotropic properties as evidenced by the hysteresis loops that appeared on rheograms.

**Table 2:** Drug content, pH, particle size and viscosity of hydrogels with different concentrations of polyamidoamine dendrimers (PAMAM)-NH_2_ generation two or three (G2 or G3).

Formulation Code	Drug Content (%)	pH	Particle Size (µm)	Viscosity ** (mPa·s)
E1	98.00 ± 1.30	8.43 ± 0.01	29.2 ± 22.41	9038.94 ± 26.28
EP1	100.95 ± 0.48	8.50 ± 0.01	28.5 ± 22.95	8810.74 ± 84.19
EP2	99.75 ± 0.13	8.53 ± 0.01	24.8 ± 17.75	6995.01 ± 14.03 *
EP3	100.00 ± 0.29	8.56 ± 0.01	22.4 ± 18.98	4583.97 ± 84.20 *
EP4	99.95 ± 0.20	8.58 ± 0.02	19.6 ± 14.25	3690.99 ± 14.04 *
EP5	102.10 ± 0.96	8.51 ± 0.01	28.9 ± 15.78	8969.49 ± 56.12
EP6	98.15 ± 0.37	8.53 ± 0.01	25.5 ± 18.68	7332.36 ± 24.67
EP7	99.20 ± 0.41	8.58 ± 0.02	23.1 ± 22.27	4752.64 ± 58.22 *
EP8	102.25 ± 0.23	8.59 ± 0.02	20.8 ± 19.06	3939.04 ± 84.20 *

* *p* < 0.05; ** viscosity was measured at shear rate 10.00 s^−1^.

Topical semi-solid formulations should possess appropriate mechanical properties, such as hardness, adhesiveness and cohesiveness. Texture analysis provides deeper insight into the vehicle properties and enables correlation to applicability of the formulation [27,28,29]. The obtained results showed a decrease in hardness, cohesiveness and adhesiveness in a PAMAM-NH_2_ concentration dependent manner (Table 3). The highest drop in the analyzed parameters was recorded in formulations containing dendrimers at concentration 6 mg/g.

**Table 3:** Mechanical properties of hydrogels with different concentrations of PAMAM-NH_2_ G2 or G3.

Formulation Code	Hardness (g)	Cohesiveness (g·s)	Adhesiveness (g·s)
E1	56.51 ± 1.26	115.91 ± 3.22	−128.25 ± 3.08
EP1	54.97 ± 2.17	111.97 ± 3.69	−132.00 ± 3.57
EP2	43.68 ± 2.28	89.87 ± 3.04	−107.72 ± 0.83 *
EP3	26.30 ± 0.43	58.59 ± 1.17	−70.43 ± 0.09 *
EP4	22.67 ± 1.03	52.02 ± 0.05	−66.56 ± 2.78 *
EP5	56.46 ± 1.42	114.75 ± 3.61	−132.24 ± 3.69
EP6	46.46 ± 1.32	95.67 ± 0.82	−111.95 ± 1.11
EP7	28.49 ± 0.55	63.60 ± 0.33	−73.44 ± 0.87 *
EP8	26.72 ± 0.11	62.38 ± 0.17	−70.77 ± 0.96 *

* *p* < 0.05.

It can be assumed that the reduction in the viscosity and in the mechanical parameters characterizing the hydrogels was the result of interaction between the positive amino groups on the dendrimer surface and hydroxyethylcellulose chains and in the consequence loosening the hydrogels structure. However, it is worth noting that a greater effect on reducing the viscosity, hardness, cohesiveness and adhesiveness of hydrogels was observed in the presence of PAMAM-NH_2_ G2 with smaller particle size. Therefore, it may be presumed that in addition to the electrostatic interactions between the polymer and PAMAM-NH_2_, noticeable decline in examined parameters can be explained by the penetration of small dendrimer molecules between polymer chains and, in consequence, weakening interactions in the polymer network [30,31].

**Figure 2:**
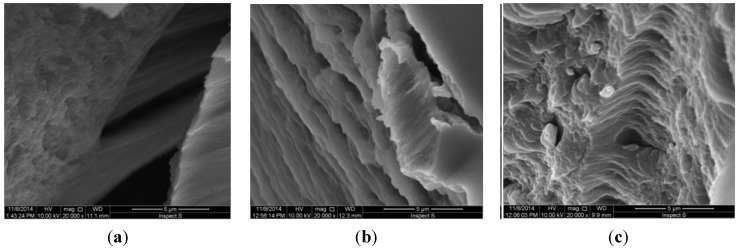
The SEM images of hydrogels: (**a**) without PAMAM-NH_2_ (E1); (**b**) with PAMAM-NH_2_ G2 3 mg/g (EP3); (**c**) with PAMAM-NH_2_ G3 3 mg/g (EP7) (magnification 20,000×).

To investigate the impact of PAMAM-NH_2_ on hydrogel surface morphology, SEM experiments were additionally performed. It was noted that hydrogel without PAMAM-NH_2_ (E1) possessed dense, uniform and regular structure with striations (Figure 2a). Characteristic changes in morphology were observed in hydrogels containing PAMAM-NH_2_ at concentration 3 mg/g (EP3 and EP7). By introducing dendrimers into hydrogel, the structure occurred visibly less compact, diversified and more porous (Figure 2b,c).

### 2.2. In Vitro Release of EM

One of the important criteria for dosage form evaluation is the analysis of the release rate of the active substance. The drug release from dermatological preparations is affected by several parameters, *i.e.*, the nature of the vehicle, the solubility of the drug substance in a vehicle and acceptor medium, drug partition coefficient between the vehicle and the water, and the viscosity of the vehicle. Generally, it is considered that the release rate increases in the following order: lipophilic ointment < cream oil/water < gel [32,33].

**Figure 3:**
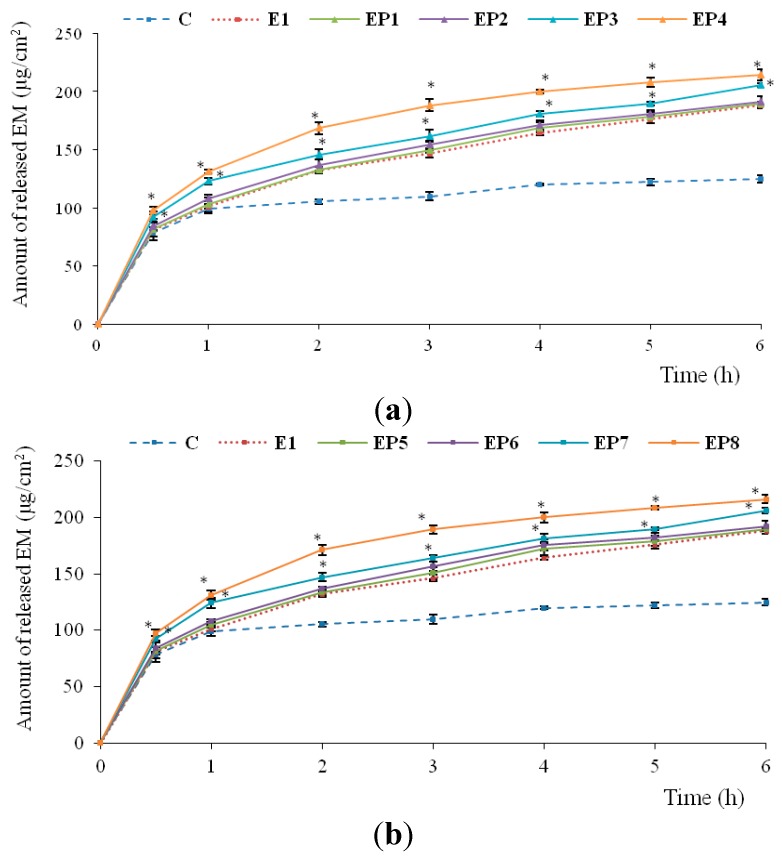
*In vitro* release of EM from hydrogels with different concentrations of (**a**) PAMAM-NH_2_ G2 or (**b**) PAMAM-NH_2_ G3 comparing with hydrogel without dendrimers (E1) and commercially available product (C). * *p* < 0.05.

*In vitro* release study showed that EM was faster released from hydrogels containing PAMAM-NH_2_ than from hydrogel without dendrimers (E1) and from commercially available product (Figure 3). The amount of released EM was PAMAM-NH_2_ concentration and generation dependent, with G3 being slightly more potent (*p* < 0.05). The largest amount of released EM was observed for hydrogels with PAMAM-NH_2_ G3 at 6 mg/g (EP8) (after 6 h, cumulative amount of released EM was 216.17 μg/cm^2^). This might be due to the improved solubility of EM caused by PAMAM-NH_2_ dendrimers. However, increasing amount of released EM may also be attributed to differences in viscosity of the investigated hydrogels. It was found that hydrogels containing higher concentrations (3 and 6 mg/g) of PAMAM-NH_2_ had a lower viscosity (even up to 2-fold in the case of PAMAM-NH_2_ G2 and G3 at concentrations 6 mg/g—EP4 and EP8, respectively). Similarly, Devarakonda *et al.* [34] demonstrated that increasing concentrations of dendrimers added to the hydrogels and significantly increased the amount of released nifedipine [34]. Another paper described the beneficial effect of PAMAM on the release profile of clotrimazole both from the hydrogels and aqueous suspensions [35].

### 2.3. Antimicrobial Activity of Hydrogels

In order to estimate the therapeutic efficacy of hydrogels containing PAMAM-NH_2_ G2 or G3, the effect of dendrimers on their antimicrobial activity was studied (Table 4). It was found that the addition of PAMAM-NH_2_ only slightly improved antimicrobial activity of hydrogels. The relatively weak influence on the improvement of antibacterial efficiency might be due to the limited possibility of lower generations of PAMAM-NH_2_ to induce changes in the bacterial cell wall permeability. Specific structure of the Gram(+) bacteria confers resistance and dendrimers molecules were probably unable to effectively interact and destabilize their plasma membranes.

**Table 4:** Antibacterial activity of hydrogels with different concentrations of PAMAM-NH_2_ G2 or G3.

Zone of Inhibition (mm)	Name of the Strain			
	*S. aureus* ATCC 29213	*S. aureus* (Clinical Strain)	*S. aureus* (Clinical Strain)	*E. faecalis* ATCC 29212
EM	37.3 ± 2.3	36.7 ± 1.2	34.5 ± 2.5	32.0 ± 0.0
C	32.0 ± 0.0	31.7 ± 1.5	31.0 ± 0.0	25.3 ± 0.6
H	5.7 ± 2.3	5.5 ± 0.7	5.0 ± 2.3	5.7 ± 0.6
E1	33.0 ± 1.0	32.3 ± 0.6	35.0 ± 1.0	30.0 ± 0.0
EP1	36.0 ± 0.0	35.3 ± 1.2	34.0 ± 0.0	31.7 ± 0.6
EP2	36.0 ± 0.0	39.0 ± 1.41	35.5 ± 0.0	31.3 ± 1.2
EP3	36.0 ± 0.0	36.0 ± 0.0	33.5 ± 0.0	30.7 ± 0.6
EP4	*-	*-	*-	*-
EP5	36.0 ± 0.0	35.7 ± 0.6	34.0 ± 0.0	30.3 ± 0.6
EP6	37.3 ± 1.2	35.3 ± 1.2	35.0 ± 0.0	30.7 ± 0.6
EP7	37.3 ± 0.6	36.3 ± 1.5	35.0 ± 0.0	30.0 ± 0.0
EP8	*-	*-	*-	*-

*- not studied due to the decrease in the hydrogels viscosity.

### 2.4. Stability Studies

To completely assess the influence of PAMAM-NH_2_ on hydrogels structure, stability studies were performed. The prepared hydrogels were subjected to three different storage conditions (4 ± 2 °C, 60% ± 5% RH, 25 ± 2 °C, 60% ± 5% RH and 40 ± 2 °C, 75% ± 5% RH) for a three month period. Hydrogels were monitored by visual estimation, evaluation of drug content, pH, particle size and viscosity. All prepared formulations were found to be stable over three months of storage and no significant changes were observed in their physical appearance, rheological properties and particles size. EM content was in the acceptable 90.0%–125.0% USP (United States Pharmacopeia) limit (data not shown) [36].

## 3. Experimental Section 

### 3.1. Chemicals and Materials

Erythromycin was received from Pharma Cosmetics (Kraków, Poland). Hydroxyethylcellulose (HEC) was obtained from A.C.E.F. (Piacenza, Italy). PAMAM-NH_2_ dendrimers G2 and G3, Tween 80, bronopol, Mueller-Hinton agar were provided by Sigma Aldrich (St. Louis, MO, USA), as were most other chemicals and buffers used. Cellulose dialysis membrane (molecular weight cut-off 3500 daltons) was received from Medicell (London, UK). Aknemycin^®^ 20 mg/g ointment used as a control was a product of Almirall, Hermal GmbH (Reinbek, Germany). All chemicals and solvents used for the study were of analytical grade. Control strains: *Staphylococcus aureus* ATCC 29213, *Enterococcus faecalis* ATCC 29212 were from American Type Culture Collection (Microbiologics^®^, St. Cloud, MN, USA). *S. aureus* clinical strains were isolated from clinical samples obtained from patients treated in departments of the University Hospital in Białystok (Poland). The bacteria were identified by the VITEK 2 GN card and the automatic system VITEK 2 (BioMerieux, Durham, NC, USA) according to manufacturer’s instructions.

### 3.2. Preparation of Hydrogels

The hydrogels were prepared by dissolving bronopol in purified water and then hydroxyethylcellulose was gradually added into the solution and stirred until homogenous mixture appeared using mechanical stirrer (IKA-Werke, Staufen, Germany). Mixing was continued until a transparent gel was received and, after that, suspensions of EM in various concentration of PAMAM-NH_2_ solutions were added (Table 1). The blending was continued to get uniform dispersion of EM in the hydrogel. The concentrations of dendrimers in hydrogels were chosen according to the previous study evaluating the *in vivo* dermal toxicity of PAMAM-NH_2_ in the rat skin model [7].

### 3.3. Physicochemical Properties of Hydrogels

#### 3.3.1. HPLC Analysis

EM content was determined after extraction of hydrogels samples in ethanol 99.9% and analysed by HPLC method in the following conditions: Waters Spherisorb^®^ 5.0 µm ODS 2, 4.6 × 250 mm, 5 µm column (Waters Corporation, Milford, MA, USA); mobile phase: acetonitrile-phosphate buffer pH 7.0 (65:35, *v*/*v*); flow rate 1.0 mL/min; detection at 195 nm; retention time 3.0 min [37,38] and retention factor (*k*) was 1.4; the standard calibration curve was linear over the range of 5–150 µg/mL (*R*^2^ = 0.999).

#### 3.3.2. Particle Size Analysis

Hydrogels samples were observed (under magnification 100×) and particle size was analyzed by using an optical microscope Motic BA 400 equipped with a camera (Moticon, Wetzlar, Germany).

#### 3.3.3. Viscosity Measurement and Determination of Rheological Properties

The viscosity was determined using Brookfield viscometer (Model RVDV-III Ultra, Brookfield Engineering Laboratories, Middlebro, MA, USA) equipped with the cone/plate type CPA52Z (plate diameter 24 mm, cone angle 3°) measuring system at temperature 25 ± 1 °C. The viscosity of hydrogels at shear rate 10.00 s^−1^ was recorded and the rheograms of the hydrogels were evaluated by plotting the experimentally obtained values of shear stress versus shear rate.

#### 3.3.4. Texture Analysis

The texture properties of prepared hydrogels were carried out using a Texture Analyser TA.XT Plus (Stable Micro System, UK) for backwards extrusion measurements. A disc (35 mm diameter) was pushed at a speed of 2 mm/s for a distance of 5 mm into the hydrogel (30 g) and redrawn. Data collection and data analysis were performed using the Texture Exponent software package. Hydrogel parameters such as hardness, cohesiveness and adhesiveness were determined from the resultant force-time plots. Hardness is defined as the maximal force required to attain a given deformation, cohesiveness as the work required to deform the hydrogel in the downwards movement of the probe, whereas adhesiveness, as the adequate work in the upwards movement of the disc, indicates the ability of the hydrogel to adhere on the disc [39,40].

#### 3.3.5. Morphology of the Hydrogels

To investigate the morphology of designed hydrogels, scanning electron microscopy (SEM) analysis was performed. After preparation, samples of the hydrogels with PAMAM-NH_2_ G2 or G3 at concentration 3 mg/g and hydrogel without PAMAM were cooled to −80 °C and dehydrated using lyophylization (Centrivap Concentrator FreeZone6; Labconco, Kansas City, MO, USA). Afterwards, the samples were sputter-coated with a thin layer of gold (6.5 nm) in an argon atmosphere (Leica EM AC 2000, Wetzlar, Germany) and imaged using SEM (Hitachi, SH 200, Tokyo, Japan).

### 3.4. In Vitro Release of EM

The release of EM was measured through natural cellulose membrane using an enhancer cell with surface area of 3.80 cm^2^. The enhancer cell consisted of a Teflon load ring, a cap, a membrane and a drug reservoir. This study was performed using the USP dissolution apparatus 2 (Agilent 708-DS, Agilent Technologies, Cary, NC, USA) with mini vessels (250 mL) and mini paddles. Samples, each of about 3 g, were placed in the enhancer cell, which was then immersed in the dissolution vessel containing 100 mL of the release medium (acetic buffer pH 5.5 with 1% Tween 80 to provide the sink conditions), previously warmed and maintained at 32 ± 0.5 °C corresponding to the skin surface temperature. Agitation was affected by mini paddles at 75 rpm and aliquots each of 2 mL were withdrawn at different time intervals (0.5, 1, 2, 3, 4, 5 and 6 h). Withdrawn samples were replaced by equal volumes of fresh release medium. The samples were assayed by HPLC method as described earlier.

### 3.5. Antimicrobial Activity of Hydrogels

The antibacterial activity of hydrogels was evaluated by the plate diffusion method on Mueller-Hinton agar. Tested microorganisms included Gram(+) bacteria: *Staphylococcus aureus* ATCC 29213, *Enterococcus faecalis* ATCC 2912 and *S. aureus* clinical strains. Clinical strains were isolated from clinical samples obtained from patients treated in the University Hospital in Białystok (Poland). The bacteria were identified by the VITEK 2 GN card and the automatic system VITEK 2 (BioMerieux, Durham, NC, USA) according to manufacturer’s instructions. Inoculation was made with a sterile broth culture diluted to match a 0.5 McFarland turbidity standard. Then Petri dishes with sterile Mueller-Hinton agar were seeded with 100 μL of the bacterial suspension. After the plates solidified at ambient temperature, a 5 mm diameter wells were cut in the inoculated agar plates and 100 mg of prepared hydrogels were placed in each well. The plates were incubated at 37 °C for 24 h. The results were recorded by measuring the zones of growth inhibition surrounding the wells. Hydrogel placebo (H), hydrogel without PAMAM (E1) as negative control, 2% solution of EM in DMSO (EM) and commercial available product Aknemycin^®^ (C) as positive control were used [14].

### 3.6. Stability Studies

The prepared hydrogels were stored for three months in sealed polyethylene containers at three different temperature and humidity conditions (4 ± 2 °C, 25 ± 2 °C and 60% ± 5% RH, 40 ± 2 °C and 75% ± 5% RH) in climatic test chambers (CTC 256, Memmert, Schwabach, Germany; KBF 115, Binder, Tuttlingen, Germany) and evaluated periodically for viscosity, pH, particle size, EM content and inspected visually for their colour, homogeneity and consistency.

### 3.7. Statistical Analysis

Results are presented as the mean ± standard deviation (SD) based on six independent experiments. Statistical analysis was done by one-way analysis of variance (ANOVA) using Statistica 10.0 software (StatSoft, Kraków, Poland). A probability level of *p* < 0.05 was considered as significant.

## 4. Conclusions

Based on the obtained results, it can be concluded that using PAMAM-NH_2_ improved the *in vitro* release of EM. Designed hydrogels were non-Newtonian systems, showing a shear-thinning behaviour with thixotropic properties. However, the addition of PAMAM-NH_2_ to hydrogels evoked only slight improvement of their antibacterial activity. It is also worth noting that PAMAM-NH_2_ at higher concentrations ≥3 mg/g influenced the viscosity and mechanical properties of designed hydrogels. This might likely be a result of their interaction with the polymer used to prepare hydrogels and suggest changes in the polymer network structure. The structure of hydrogels in the presence of PAMAM-NH_2_ became visibly less compact and porous.
